# Effectiveness of mindfulness-based interventions on well-being and work-related stress in the financial sector: a systematic review and meta-analysis protocol

**DOI:** 10.1186/s13643-022-01956-x

**Published:** 2022-04-27

**Authors:** Tantri Keerthi Dinesh, Ankitha Shetty, Vijay Shree Dhyani, Shwetha T.S, Komal Jenifer Dsouza

**Affiliations:** 1 Department of Commerce, Manipal Academy of Higher Education, Manipal, Karnataka India; 2 Public Health Evidence South Asia (PHESA), Prasanna School of Public Health (PSPH), Manipal Academy of Higher Education, Manipal, Karnataka India; 3 Department of Clinical Psychology, Manipal Academy of Higher Education, Manipal, Karnataka India

**Keywords:** Work-related stress, Mindfulness-based interventions, Well-being, Finance sector, MBIs

## Abstract

**Background:**

Work-related stress is a common phenomenon, often noticed in the employees of the finance sector. It mirrors counter effects on the wellness of employees, their mental well-being, and physical health. Mindfulness-based interventions (MBIs) raise awareness and attention to the present moment experiences by adopting coping skills. It is necessary to promote employee well-being and reduce work-related stress; hence, the need arises to associate between the level of mindfulness, employee well-being, and work-related stress. A systematic review on the effectiveness of MBIs in the finance sector is necessary to facilitate evidence for the future utility to reduce work-related stress and promote employee well-being.

**Methods:**

In this review, randomized controlled trials, non-randomized control trials, cohort, and cross-sectional and case-control studies that assess the effectiveness of MBIs on the employees in the finance sector will be considered. We propose to perform a literature search which will be conducted from the years 2000 to 2021 on CINAHL, Cochrane Library, ProQuest, PubMed, Scopus, and Web of Science. The search terms will include controlled and accessible terms such as mindfulness-based interventions, mindfulness training, workplace, employees, workers, well-being, employee wellness, occupational health, and finance sector. The outcomes will include the effect on employee well-being and reduction in work-related stress. Two researchers will independently conduct the screening and data extraction and assess the risk of bias. Based on the availability of data, a meta-analysis will also be performed. This protocol follows the Preferred Reporting Items for Systematic reviews and Meta-Analysis-Protocol (PRISMA-P) guidelines. “Assessing the Methodological Quality of Systematic Reviews” will be used to assess the quality of this review.

**Discussion:**

The review attempts to methodically analyse the effectiveness of MBIs among finance sector employees. It will foster to facilitate a detailed description and evidence-based overview of the effectiveness of MBIs on improving work-related stress, mindful awareness, and employee wellness and well-being in employees in the finance sector. The current study will provide an evidence base to researchers, academicians, and practitioners in the selection of mindfulness-based therapies for employees in the finance sector.

**Systematic review registration:**

PROSPERO 2021 CRD42021249782

**Supplementary Information:**

The online version contains supplementary material available at 10.1186/s13643-022-01956-x.

## Introduction

Globalization is driving off the fast-paced and everchanging working conditions, where strategies for alleviating employee stress play a vital role to ensure the mental well-being of workers [1] that improves the productivity of organizations [2]. Work-related stress is the most inherent psychological outcome that leaps beyond the surface pressure in any occupation [3] where finance sector is no exception and is greatly affected in-depth.

The finance sector plays an indispensable role in uplifting the economy which may be affected by high diversifications, risk management, and also due to employee attrition resulting from work-related stress [4]. Various financial service sectors, viz., banks, insurance companies, stock markets, and real estate brokers and agents, are booming in the global economy, which is not an exception for its employees are also experiencing the work-related stress due to tight deadlines and work pressures [5]. Stress in the financial service arena comprises emotional, cognitive, behavioural, and physiological reactions to noxious and aversive aspects prevailing in a turbulent work atmosphere. This environment in the finance sector is because of high levels of distress of not coping due to target pressures, strict deadlines, and excessive workload ascribed by time squeeze, lengthy working hours, intensified workload, shortage of skills, and workforce globalization [6]. Hence, it is evident that the workforce in the finance sector faces proliferating levels of stress which needs to be given utmost attention. It is the need of the hour to facilitate a positive mindset among the employees in the financial service sector to improve productivity and enable them to work efficiently [7]. There are abundant sources of stress management techniques and training to enhance the well-being and wellness of the employees wherein an essential tool to balance the mind and body is the mindfulness technique [8]. Mindfulness interventions aim at developing a mindful approach to enhance the quality of life, to reduce distress, to prevent relapse in the employees, and also to encourage “greater awareness of present moment and experience” [9]. These mindfulness interventions and techniques may differ in boundless ways that will help employees in the finance sector to cultivate a habit of self-awareness, which embarks attending to moment-to-moment experience consciously that will enhance the employee well-being [10]. Employees learn a lot about mindful focus through different experiential and introspective exercises during the interventions, which comforts and succours them to pay attention to their ongoing cognitive, emotional, and sensory experiences without judging upon or elaborating any portion of their experience [11]. Incorporating mindfulness in the finance sector with intervention techniques would contribute to enhancing self-regulation and awareness and improving the well-being and wellness that will foster the sustainability of the employees [12]. Furthermore, it is the need of the hour to incorporate deliberate strategies for improving employee well-being and wellness mechanisms that will contribute to stress management. Mindfulness interventions are proven to be impactful in the finance sector as the stress levels in this domain are alarmingly high and are prone to backbreaking. Hence, it is evident that the workforce in the finance sector faces increasing levels of stress which need to be addressed presently.

### Literature overview

Finance service work-related stress also results in increased burnout defined as “a syndrome of depersonalization, emotional exhaustion, and a sense of low personal accomplishment”, which can be mitigated through mindfulness interventions. Mindfulness is defined as “receptive attention to and awareness of present events and experience” [13]. The significant portion of meditation and mindfulness-based workplace intervention for reducing stress and improving wellness is well documented in health care professionals [14] and is slowly being harnessed in an organizational setup [15].

MBIs at the workplace are short interventions (precisely six to eight sessions) practised in a group that incorporates mindfulness and meditation exercises with certain principles contributing to an employee’s wellness [16]. “Mindfulness-based cognitive therapy” (MBCT) and “mindfulness-based stress reduction” (MBSR) are the main intervention providers under MBIs [17]. MBIs also comprise exercises involving mindfulness training as a portion of the intervention programme, which may encompass “acceptance and commitment therapy” (ACT) [12], “compassion-focused therapy” (CFT), integrative body-mind training, “dialectical behaviour therapy” (DBT) [18], and “cognitive behavioural stress management” (CBSM) which are proving to be beneficial for treating work-related stress [19].

These programmes have been incorporated increasingly to decrease workplace stress as well as improve employees’ well-being [20]. The ultimate purpose of work-related MBIs is to help to increase non-judgemental awareness with moment-to-moment attention towards the present internal as well as external experiences [21]. These interventions contribute to the employees’ well-being or wellness by adopting a health promotion programme that will maximize the likelihood of achieving positive results at the workplace [22].

Well-being or wellness is defined as “the ability to carry out daily tasks with a positive vitality”, where definitions of wellness as well-being are used interchangeably, wellness being a more holistic concept [23]. The World Health Organization defines well-being as “a state of complete physical, mental and social wellbeing and not merely the absence of disease or infirmity”. Wellness and well-being are the two terms used ubiquitously in day-to-day communications, health practice, and literature [24]. Literature highlights that the value of health is included in both wellness and well-being, but well-being additionally encompasses welfare, happiness, and comfort. Research has expanded the scope of wellness and well-being that embraces health and other beneficial attributes [25].

Despite the growing concern in the finance sector, literature showcases that very little research on work-related stress has been executed so far [26]. Much of the research that has been carried out on job stress has enthralled domains such as health, education, and hotel but not banking, [27] insurance, [28] stock market agencies, and real estate, which is considered as one of the most traumatic and pressured occupations in the financial sector [29].

Systematic reviews and meta-analyses support a comprehensive and extensive assessment of empirical efficacy, which report synthesized effect sizes based on randomized control trials [30]. The exponential extension in the popularity showcases the application of intervention research as the number of publications on randomized control trials (RCTs) has been multiplying from the years 2016 to 2020 [31]. Keeping in consideration the various requisites of the employees in finance sectors, several modifications have been made to manualize MBIs by developing curricula through physical exercise, yoga, and emotion regulation which will enhance in synergizing the benefits of being mindful [32]. Thus, to consolidate, the evidence on the effectiveness of MBIs is much needed for its applicability and usefulness in the finance sector. An emergent need of building a review on the knowledge possessed regarding mindfulness interventions in the finance sector is to be focussed with the support of published contemporary research.

### The rationale of the study

As underlined in the overview of the mentioned literature, examining the effectiveness of MBIs in the finance sector by considering employee well-being and wellness is expanding steadily [33]. However, presently, there is limited knowledge on mindfulness intervention initiatives that could be taught to counteract work-related stress and its detrimental effects on health and well-being in the employees working in the finance sector. This systematic review focuses on addressing the gap by identifying, synthesizing, and evaluating the significant initiatives which could be taken to reduce, eliminate, or mitigate work-related stress through mindfulness interventions at the workplace, which could foster benefits in improving mental health, well-being, or wellness.

The present systematic review focuses on two main objectives.

1. To assess and synthesize the effectiveness of mindfulness-based interventions on work-related stress in the financial sector
2. To examine the effectiveness of mindfulness-based intervention in improving employee well-being or wellness in the financial sector

## Methods

### Review registration and reporting—registered under PROSPERO 2021 CRD42021249782

This review aims to consolidate evidence on the effectiveness of MBIs in the finance sector. The current protocol is reported under the requirements of “Preferred Reporting Items for Systematic Reviews and Meta-Analysis Protocols” (PRISMA-P) [34], provided in Supplementary data file 1. Standard methods shall be considered for search, screening, extraction of data, data synthesis, and meta-analysis. The “Participants, Interventions, Comparisons, and Outcomes” (PICO) strategy will be inculcated at each stage in accordance with the scope of the present review article.

### Eligibility criteria

The review will include studies publications which (a) have a focus on MBIs and other interventions based on mindfulness practices, (b) report empirical results pertaining to work-related stress and employee wellness/well-being, (c) are based in a workplace specifically the finance sector, (d) are in English, and (e) are published between 2000 till date. There are no exclusion criteria based on geography as studies from around the globe will be included. The inclusion-exclusion criteria are provided in Table 1.

**Table 1:** Inclusion and exclusion criteria

Criteria	Inclusion criteria	Exclusion criteria
**Population**	Employees in finance sectors—banking, insurance industry, real estates, stockbrokers, managers in finance departments of call centres	Employees working in other sectors like nursing, hotel management, transport, physicians, education.
**Intervention**	Mindfulness-based interventions	Interventions not contributing to mindfulness will be excluded.
**Outcome**	Well-being or wellness of employees	Studies not reporting to wellness or well-being of employees in the finance sector will be excluded.
**Study design**	Randomized controlled trials (RCTs), non-RCTs, observational designs (cross-sectional, case-control, and cohort studies)	Systematic reviews, meta-analyses, clinical case studies, qualitative studies, editors’ letters and editorials.
**Time frame**	2000 to present	Prior to the year 2000 will be excluded.

### Participants

Participants above 18 years employed within an organization in the finance sector and must have undergone MBIs to reduce work-related stress will be included. The finance sector includes banks, insurance companies, financial institutions, real estate, financial call centres, and stock markets.

### Intervention

The study aims at the effectiveness of MBIs related to employee well-being, wellness, and work-related stress. Other interventions related to mindfulness practices that do not fall under the category of MBIs but are related to employee wellness and work-related stress will also be included. MBIs including mindfulness-based stress reduction (MBSR) [35], mindfulness-based cognitive therapy (MBCT) [36], dialectical behaviour therapy (DBT) [12], acceptance and commitment therapy (ACT) [17], integrated body-mind training, and compassion-focused therapy [37] incorporated in the finance sector will be included in the study. It may also include simple stress-relieving activities like yoga, anchor points exercise, mindful breathing, mindful eating, mindful walking, nuclear exercise, or any other intervention at the workplace specifically the finance sector [28]. The measures of interventions incorporated by the experimental group should be mindfulness interventions or interventions linked to combined treatment methods promoting the well-being and wellness of an employee in the finance sector. The control group is the one that receives treatment different from the experimental group, such as health education or any semi-structured talks and conversations about work-related stress. The waitlist control group is those groups where no intervention is executed in the control group [38].

### Outcome measures

The primary outcomes will include outcomes related to employees’ wellness or well-being and work-related stress as an effect of the MBIs. A general imprecision can be drawn in the adherence to mindfulness interventions at the workplace with improvements in the mental well-being of the employees in the finance sector. These outcomes include heightened present moment awareness at the workplace, greater acceptance of employees’ work situations, and increased ability to cope and remain calm in work situations that are stressful or challenging. Apart from this, it also contributes to developing healthy relationships at the workplace and better adaptability. Outcome tools may include scales such as the 40-item Occupational Stress Indicator (OSI-2) [39], Psychological Stress Measure (PSM-9) [11], the 12-item Cognitive and Affective Mindfulness Scale-Revised (CAMS-R) [40], Perceived Stress Scale of 10 items [41], Bradburn’s Affect Experience Index (AEI) [42], Psychological Distress Manifestation Scale (PDMS) [12], Shortened Warwick-Edinburgh Mental Wellbeing Scale (SWEMWB), and Five-Facet Mindfulness Scale (39-items) [43].

### Study design

The review will include RCTs, non-RCTs, observational designs (cross-sectional, case-control, and cohort studies) that have a focus on MBIs in the finance sector to promote well-being and reduce work-related stress. The language will be restricted to English. Duplicate publications and protocols on RCTs, letters, editorials, commentaries, and viewpoints will be excluded. With RCTs, both active or waitlist control conditions will be considered. Systematic reviews, meta-analyses, clinical case studies, qualitative studies, editors’ letters and editorials will be excluded from the study.

### Reported software and steps for conducting a meta-analysis

Extracted data will be entered into Cochrane Collaboration’s Review Manager (RevMan V.5.3).

In case of insufficient data for calculating the effect sizes or determining the methodological quality of the original study, the authors of the respective studies will be contacted for clarification.

### Search strategy

The literature search will be performed from the year 2000 till the present on CINAHL, Cochrane Library, ProQuest, PubMed, Scopus, and Web of Science. The search terms will include controlled and free terms such as mindfulness-based interventions, mindfulness training, meditation, workplace, employees, workers, well-being, employee wellness, occupational health, finance sector, finance employees. A comprehensive search strategy using indexed descriptors and keywords will be developed. Initially, the search will be conducted in PubMed and modified later for various databases. The initial preliminary search conducted on PubMed is provided in Supplementary data file 2. Reference of the included studies and a search for relevant reference lists of previously published reviews will also be conducted to get other relevant articles. An example of the search strategy is depicted in Table 2, and search results on CINAHL, ProQuest, PubMed, Scopus, and Web of Science are provided in Supplementary data file 3.

**Table 2:** Search strategy

Concept	Alternative words
Population (finance sector employees)	“Finance” OR “Banking” OR “Call center” OR “Real estate” OR “Insurance”
Intervention (MBIs)	“Mindfulness-Based Interventions” OR “MBIs” OR “Mindful practices” OR “Dialectical Behaviour Therapy” OR DBT OR “Mindfulness Training” OR “Mindfulness-Based Stress Reduction” OR “MBSR” OR “MBCT” OR “Mindfulness-Based Cognitive Therapy” OR “ACT” OR “Acceptance and Commitment Therapy” OR “Compassion Focused Therapy”
Outcomes (well-being)	“Work-related stress” OR “wellbeing” OR “wellness” OR “Occupational stress” OR “Stress” OR “Occupational Health” OR “Occupational wellbeing”

### Data collection and management

Data will be managed using Zotero 6.0 version. Microsoft Excel will be used for screening and data extraction.

### Study selection

The search results acquired from electronic databases and hand searches will be merged through a reference management software called Zotero 6.0 version. All titles and abstracts will be screened by two researchers independently against the set inclusion/exclusion criteria to remove irrelevant articles. A senior reviewer will be involved in resolving the disagreements on inclusion. The articles included for full-text screening shall be retrieved and examined independently by two reviewers whereby any kind of disagreement will be discussed till consensus and a final decision will be taken in discussion with a third reviewer. Detailed records of the outcomes at every stage, including all the rejected articles with reasons for rejection at the full-text stage, will be reported and depicted in the PRISMA flow chart [44]. Studies that fail to fulfil the eligibility criteria will be excluded from the review.

### Data extraction

The studies included at the full-text stage will be carried forward for data extraction. Extraction of data will be independently performed by two researchers using the piloted data extraction form on Microsoft Excel. Disagreements will be discussed between two reviewers and upon disagreement, a third reviewer/senior reviewer will examine the extracted data and give a final decision. The major extraction content will include study characteristics, methodological details, and outcome measures along with effect sizes. The information on authors, publication year, region, participants (gender and age), study design, intervention methods, duration of the interventions, evaluation and assessment methods, outcomes, and significant findings along with follow-up durations will be extracted.

### Dealing with missing data

In case of any missing data, the corresponding author of the respective research paper will be contacted for further information. If there is a delay or no reply from the concerned author, the co-authors or the corresponding author of the research study will be contacted for further details and clarifications.

### Risk of bias assessment

The quality assessments will be done by two reviewers independently. Discrepancies if any will be resolved in a discussion with a third reviewer. The quality of the included studies will be assessed through different tools based on the study design, viz, randomized control trials (RCTs): Cochrane Risk-of-Bias (RoB 2) [45]; non-RCTs: Risk of Bias in Non-randomized Studies - of Interventions (ROBINS-I tool) [45]; cohort and case-control studies: Newcastle-Ottawa Scale (NOS) [46]; and cross-sectional studies: an adapted version of NOS.

### Quality assessment of systematic review

AMSTAR 2 will be used as the methodological guidance [47] in the current study. The quality of the evidence will be assessed using the Grading of Recommendations, Assessment, Development and Evaluation (GRADE) approach [48].

### Data analysis

#### Data synthesis

Based on the availability of data, a quantitative synthesis of the findings in the form of a meta-analysis will be provided. The 95% confidence interval around effect sizes will be reported. Odds ratio (OR) and relative risk (RR) will be extracted for summarizing the categorical data, whereas summarization of continuous data will be done with a standardized mean difference. Summary measures will be pooled based on the study design. As considerable heterogeneity among studies is expected, a random-effects model will be used. Pooled estimates will be reported with 95% CIs in a forest plot. Assessment of the heterogeneity of effect sizes will be done by using the *I*^2^ statistic. According to the Cochrane handbook, *I*^2^ values will be interpreted as unimportant (*I*^2^ < 40%), moderate (30–60%), substantial (50–90%), or considerable heterogeneity (75–100%). If the included studies are sufficiently homogenous (*I*^2^ ≤ 60%) or heterogeneity is sufficiently reduced by the prespecified differentiation into subgroups, we will use a random-effects model to meta-analyse the primary studies. In the case of RCTs, we will be calculating the effect sizes for MBIs at the workplace using the standardized intervention-control difference (between-group comparison) with RCTs and the standardized post/preintervention difference (within-group comparison) with non-randomized control trials. We will be assessing the different types of non-randomized control interventions. Alongside, we will also be assessing the observational studies which may be categorized as case-control, concurrent (prospective) cohort, and nonconcurrent (retrospective) cohort.

#### Sensitivity and subgroup analysis

The source of heterogeneity for meta-analysis shall be explored with sensitivity and subgroup analysis. RCTs, non-RCTs, and observational studies will be contrasted to evaluate potential differences in the estimation of MBIs’ effectiveness due to different study designs and study settings. Publication bias will be assessed if included studies are more than 10 using a funnel plot [49].

#### Narrative synthesis

In case of the non-availability of data for quantitative synthesis, the data will be summarized narratively for review outcomes especially if the heterogeneity of the included studies is large.

#### Reporting of the systematic review and meta-analysis

The PRISMA guidelines will be used for reporting the systematic literature review and meta-analysis [34].

## Discussion

This will be the first systematic review to consolidate the effectiveness of MBIs among employees of the finance sectors on work-related stress and improving wellness wherein it is not for any update of a previous systematic review. Also, this protocol does not represent an amendment of a previously completed or published protocol.

Extensive and considerable search strategies and inclusion criteria will be contemplated in the present review, describing a systematic review and meta-analysis of the obtainable evidence where detailed retrieval strategies are being formulated and there are no possibilities of including unpublished trials. The source of heterogeneity for meta-analysis shall be explored with sensitivity and subgroup analysis. A random-effects model will be incorporated with the expectation of considerable heterogeneity amidst the studies. Generation of a forest plot would be implemented where pooled estimates will be reported with 95% CIs. There will be no restriction with respect to geographical region and will consider studies globally and we will not be including case studies and qualitative studies. Occupational and workplace-related stress is hard to define, making the inclusion criteria subjective.

The review attempts to analyse the effectiveness of MBIs among finance sector employees methodically. It will facilitate a detailed description and evidence-based overview of the effectiveness of MBIs on improving work-related stress, mindful awareness, and employee wellness and well-being in employees in the finance sector. An evidence base will be provided by the current study to researchers, academicians, and practitioners in the selection of mindfulness-based therapies for employees in the finance sector.

## Supplementary Information

**Additional file 1: Supplementary data file 1**: PRISMA-P 2015 Checklist.

**Additional file 2: Supplementary data file 2**: PubMed Search.

**Additional file 3: Supplementary data file 3**: Search Strategy.

## Data Availability

Not applicable.

## Abbreviations

ACT: Acceptance and commitment therapy
AEI: Bradburn’s Affect Experience Index
AMSTAR 2: Assessing the Methodological Quality of Systematic Reviews
CAMS-R: Cognitive and Affective Mindfulness Scale-Revised
CI: Confidence interval
DBT: Dialectical behaviour therapy
HQ: Health questionnaire
MBCT: Mindfulness-based cognitive therapy
MBI: Mindfulness-based interventions
MBSR: Mindfulness-based stress reduction
NOS: Newcastle-Ottawa Scale
OR: Odds ratio
OSI-2: Occupational Stress Indicator
PDMS: Psychological Distress Manifestation Scale
PICO: Participants, Interventions, Comparisons, and Outcomes
PRISMA-P: Preferred Reporting Items for Systematic Reviews and Meta-Analysis Protocols
PSM-9: Psychological Stress Measure
PSS: Perceived Stress Scale
RCT: Randomized control trial
ROB: Risk of bias
ROBINS-I: Risk of Bias in Non-randomized Studies - of Interventions
RR: Relative risk
SWEMWB: Shortened Warwick-Edinburgh Mental Wellbeing Scale
