# Supplementary material for: Effectiveness of mindfulness-based interventions on well-being and work-related stress in the financial sector: a systematic review and meta-analysis protocol
Source: Syst Rev. 2022 Apr 27;11:79. doi: 10.1186/s13643-022-01956-x (PMC9047319; doi:10.1186/s13643-022-01956-x)
Supplement: Supplementary file 2 — Additional file 2: Supplementary data file 2: PubMed Search. [file 13643_2022_1956_MOESM2_ESM.docx]

| **Search** | **Query** | **Results** |
| --- | --- | --- |
| #5 | Search: **(((("Mindfulness-based interventions"[Title/Abstract] OR "MBIs"[Title/Abstract] OR "Mindful practices"[Title/Abstract] OR "Dialectical Behaviour Therapy"[Title/Abstract] OR DBT[Title/Abstract] OR "Mindfulness Training"[Title/Abstract] OR "Mindfulness-Based Stress Reduction"[Title/Abstract] OR "MBSR"[Title/Abstract] OR "MBCT"[Title/Abstract] OR "Mindfulness-Based Cognitive Therapy"[Title/Abstract] OR "ACT"[Title/Abstract] OR "Compassion Focused Therapy"[Title/Abstract])) OR ("Acceptance and Commitment Theory"[Title/Abstract] OR "ACT"[Title/Abstract])) AND ("Work related stress"[Title/Abstract] OR "wellbeing"[Title/Abstract] OR "wellness"[Title/Abstract] OR "Occupational stress"[Title/Abstract] OR "Stress"[Title/Abstract] OR "Occupational Health"[Title/Abstract] OR "Occupational wellbeing"[Title/Abstract])) AND ("Finance"[Title/Abstract] OR "Banking"[Title/Abstract] OR "Call centre"[Title/Abstract] OR "Real estate"[Title/Abstract] OR "Insurance"[Title/Abstract])**  ("Mindfulness-based interventions"[Title/Abstract] OR "MBIs"[Title/Abstract] OR "Mindful practices"[Title/Abstract] OR "Dialectical Behaviour Therapy"[Title/Abstract] OR "DBT"[Title/Abstract] OR "Mindfulness Training"[Title/Abstract] OR "Mindfulness-Based Stress Reduction"[Title/Abstract] OR "MBSR"[Title/Abstract] OR "MBCT"[Title/Abstract] OR "Mindfulness-Based Cognitive Therapy"[Title/Abstract] OR "ACT"[Title/Abstract] OR "Compassion Focused Therapy"[Title/Abstract] OR ("Acceptance and Commitment Theory"[Title/Abstract] OR "ACT"[Title/Abstract])) AND ("Work related stress"[Title/Abstract] OR "wellbeing"[Title/Abstract] OR "wellness"[Title/Abstract] OR "Occupational stress"[Title/Abstract] OR "Stress"[Title/Abstract] OR "Occupational Health"[Title/Abstract] OR "Occupational wellbeing"[Title/Abstract]) AND ("Finance"[Title/Abstract] OR "Banking"[Title/Abstract] OR "Call centre"[Title/Abstract] OR "Real estate"[Title/Abstract] OR "Insurance"[Title/Abstract]) | [170](https://pubmed.ncbi.nlm.nih.gov/?term=%28%28%28%28%22Mindfulness-based+interventions%22%5BTitle%2FAbstract%5D+OR+%22MBIs%22%5BTitle%2FAbstract%5D+OR+%22Mindful+practices%22%5BTitle%2FAbstract%5D+OR+%22Dialectical+Behaviour+Therapy%22%5BTitle%2FAbstract%5D+OR+DBT%5BTitle%2FAbstract%5D+OR+%22Mindfulness+Training%22%5BTitle%2FAbstract%5D+OR+%22Mindfulness-Based+Stress+Reduction%22%5BTitle%2FAbstract%5D+OR+%22MBSR%22%5BTitle%2FAbstract%5D+OR+%22MBCT%22%5BTitle%2FAbstract%5D+OR+%22Mindfulness-Based+Cognitive+Therapy%22%5BTitle%2FAbstract%5D+OR+%22ACT%22%5BTitle%2FAbstract%5D+OR+%22Compassion+Focused+Therapy%22%5BTitle%2FAbstract%5D%29%29+OR+%28%22Acceptance+and+Commitment+Theory%22%5BTitle%2FAbstract%5D+OR+%22ACT%22%5BTitle%2FAbstract%5D%29%29+AND+%28%22Work+related+stress%22%5BTitle%2FAbstract%5D+OR+%22wellbeing%22%5BTitle%2FAbstract%5D+OR+%22wellness%22%5BTitle%2FAbstract%5D+OR+%22Occupational+stress%22%5BTitle%2FAbstract%5D+OR+%22Stress%22%5BTitle%2FAbstract%5D+OR+%22Occupational+Health%22%5BTitle%2FAbstract%5D+OR+%22Occupational+wellbeing%22%5BTitle%2FAbstract%5D%29%29+AND+%28%22Finance%22%5BTitle%2FAbstract%5D+OR+%22Banking%22%5BTitle%2FAbstract%5D+OR+%22Call+centre%22%5BTitle%2FAbstract%5D+OR+%22Real+estate%22%5BTitle%2FAbstract%5D+OR+%22Insurance%22%5BTitle%2FAbstract%5D%29&sort=) |
| #4 | Search: **"Finance"[Title/Abstract] OR "Banking"[Title/Abstract] OR "Call centre"[Title/Abstract] OR "Real estate"[Title/Abstract] OR "Insurance"[Title/Abstract]**  "Finance"[Title/Abstract] OR "Banking"[Title/Abstract] OR "Call centre"[Title/Abstract] OR "Real estate"[Title/Abstract] OR "Insurance"[Title/Abstract] | [108,463](https://pubmed.ncbi.nlm.nih.gov/?term=%E2%80%9CFinance%E2%80%9D%5BTitle%2FAbstract%5D+OR+%E2%80%9CBanking%E2%80%9D%5BTitle%2FAbstract%5D+OR+%E2%80%9CCall+centre%E2%80%9D%5BTitle%2FAbstract%5D+OR+%E2%80%9CReal+estate%E2%80%9D%5BTitle%2FAbstract%5D+OR+%E2%80%9CInsurance%E2%80%9D%5BTitle%2FAbstract%5D&ac=no&sort=relevance) |
| #3 | Search: **"Work related stress"[Title/Abstract] OR "wellbeing"[Title/Abstract] OR "wellness"[Title/Abstract] OR "Occupational stress"[Title/Abstract] OR "Stress"[Title/Abstract] OR "Occupational Health"[Title/Abstract] OR "Occupational wellbeing"[Title/Abstract]**  "Work related stress"[Title/Abstract] OR "wellbeing"[Title/Abstract] OR "wellness"[Title/Abstract] OR "Occupational stress"[Title/Abstract] OR "Stress"[Title/Abstract] OR "Occupational Health"[Title/Abstract] OR "Occupational wellbeing"[Title/Abstract] | [943,223](https://pubmed.ncbi.nlm.nih.gov/?term=%E2%80%9CWork+related+stress%E2%80%9D%5BTitle%2FAbstract%5D+OR+%E2%80%9Cwellbeing%E2%80%9D%5BTitle%2FAbstract%5D+OR+%E2%80%9Cwellness%E2%80%9D%5BTitle%2FAbstract%5D+OR+%E2%80%9COccupational+stress%E2%80%9D%5BTitle%2FAbstract%5D+OR+%E2%80%9CStress%E2%80%9D%5BTitle%2FAbstract%5D+OR+%E2%80%9COccupational+Health%E2%80%9D%5BTitle%2FAbstract%5D+OR+%E2%80%9COccupational+wellbeing%E2%80%9D%5BTitle%2FAbstract%5D&ac=no&sort=relevance) |
| #2 | Search: **"Acceptance and Commitment Theory"[Title/Abstract] OR "ACT"[Title/Abstract]**  "Acceptance and Commitment Theory"[Title/Abstract] OR "ACT"[Title/Abstract] | [293,074](https://pubmed.ncbi.nlm.nih.gov/?term=%22Acceptance+and+Commitment+Theory%22%5BTitle%2FAbstract%5D+OR+%22ACT%22%5BTitle%2FAbstract%5D&ac=no&sort=relevance) |
| #1 | Search: **"Mindfulness-based interventions"[Title/Abstract] OR "MBIs"[Title/Abstract] OR "Mindful practices"[Title/Abstract] OR "Dialectical Behaviour Therapy"[Title/Abstract] OR DBT[Title/Abstract] OR "Mindfulness Training"[Title/Abstract] OR "Mindfulness-Based Stress Reduction"[Title/Abstract] OR "MBSR"[Title/Abstract] OR "MBCT"[Title/Abstract] OR "Mindfulness-Based Cognitive Therapy"[Title/Abstract] OR "ACT"[Title/Abstract] OR "Compassion Focused Therapy"[Title/Abstract]**  "Mindfulness-based interventions"[Title/Abstract] OR "MBIs"[Title/Abstract] OR "Mindful practices"[Title/Abstract] OR "Dialectical Behaviour Therapy"[Title/Abstract] OR "DBT"[Title/Abstract] OR "Mindfulness Training"[Title/Abstract] OR "Mindfulness-Based Stress Reduction"[Title/Abstract] OR "MBSR"[Title/Abstract] OR "MBCT"[Title/Abstract] OR "Mindfulness-Based Cognitive Therapy"[Title/Abstract] OR "ACT"[Title/Abstract] OR "Compassion Focused Therapy"[Title/Abstract] | [299,275](https://pubmed.ncbi.nlm.nih.gov/?term=%E2%80%9CMindfulness-based+interventions%E2%80%9D%5BTitle%2FAbstract%5D+OR+%E2%80%9CMBIs%E2%80%9D%5BTitle%2FAbstract%5D+OR+%E2%80%9CMindful+practices%E2%80%9D%5BTitle%2FAbstract%5D+OR+%E2%80%9CDialectical+Behaviour+Therapy%E2%80%9D%5BTitle%2FAbstract%5D+OR+DBT%5BTitle%2FAbstract%5D+OR+%E2%80%9CMindfulness+Training%E2%80%9D%5BTitle%2FAbstract%5D+OR+%E2%80%9CMindfulness-Based+Stress+Reduction%E2%80%9D%5BTitle%2FAbstract%5D+OR+%E2%80%9CMBSR%E2%80%9D%5BTitle%2FAbstract%5D+OR+%E2%80%9CMBCT%E2%80%9D%5BTitle%2FAbstract%5D+OR++%E2%80%9CMindfulness-Based+Cognitive+Therapy%E2%80%9D%5BTitle%2FAbstract%5D+OR+%E2%80%9CACT%E2%80%9D%5BTitle%2FAbstract%5D+OR+%E2%80%9CCompassion+Focused+Therapy%E2%80%9D%5BTitle%2FAbstract%5D&ac=no&sort=relevance) |
