# Supplementary material for: Effectiveness of mindfulness-based interventions on well-being and work-related stress in the financial sector: a systematic review and meta-analysis protocol
Source: Syst Rev. 2022 Apr 27;11:79. doi: 10.1186/s13643-022-01956-x (PMC9047319; doi:10.1186/s13643-022-01956-x)
Supplement: Supplementary file 3 — Additional file 3: Supplementary data file 3: Search Strategy. [file 13643_2022_1956_MOESM3_ESM.docx]

| **Serial No.** | **DataBase** | **Search String** | **Date of Search** | **Results** |
| --- | --- | --- | --- | --- |
| 1 | Scopus | ALL ( mindfulness AND based AND interventions OR mbis OR mindfulness AND practices OR dialectical AND behaviour AND therapy OR acceptance AND commitment AND therapy OR mindfulness AND training AND work AND related AND stress OR stress OR occupational AND stress OR wellbeing OR wellness OR occupational AND health OR occupational AND wellbeing ) AND ( LIMIT-TO ( LANGUAGE , "English" ) ) | 4th March 2022 | 756 results |
| 2 | WOS | (ALL=(Mindfulness based interventions OR MBIs OR Mindful Practices OR Mindfulness Training)) AND ALL=(Work-Related Stress OR Stress OR Occupational Stress OR Wellness OR Wellbeing OR Occupational Health OR Occupational Wellbeing ) and 2000 or 2001 or 2002 or 2003 or 2004 or 2005 or 2006 or 2007 or 2008 or 2009 or 2010 or 2011 or 2012 or 2013 or 2022 or 2021 or 2020 or 2019 or 2018 or 2017 or 2016 or 2015 or 2014 (Publication Years) | 4th March 2022 | 5026 results |
| 3 | PubMed | Search: ((((Mindfulness Based Interventions) OR (MBI[Title/Abstract])) OR (Mindful Practices[Title/Abstract])) OR (Mindful Training[Title/Abstract])) AND (((((((Work-Related Stress[Title/Abstract]) OR (Stress[Title/Abstract])) OR (Occupational Stress[Title/Abstract])) OR (Wellness[Title/Abstract])) OR (Wellbeing[Title/Abstract])) OR (Occupational Health[Title/Abstract])) OR (Occupational Wellbeing[Title/Abstract])) Filters: English, from 2000/1/1 - 2020/1/1 (((("mind s"[All Fields] OR "minded"[All Fields] OR "Mindful"[All Fields] OR "mindfulness"[MeSH Terms] OR "mindfulness"[All Fields] OR "minding"[All Fields] OR "minds"[All Fields]) AND ("based"[All Fields] OR "basing"[All Fields]) AND ("intervention s"[All Fields] OR "interventions"[All Fields] OR "interventive"[All Fields] OR "methods"[MeSH Terms] OR "methods"[All Fields] OR "intervention"[All Fields] OR "interventional"[All Fields])) OR "MBI"[Title/Abstract] OR "mindful practices"[Title/Abstract] OR "mindful training"[Title/Abstract]) AND ("work related stress"[Title/Abstract] OR "Stress"[Title/Abstract] OR "occupational stress"[Title/Abstract] OR "Wellness"[Title/Abstract] OR "Wellbeing"[Title/Abstract] OR "occupational health"[Title/Abstract] OR "occupational wellbeing"[Title/Abstract])) AND ((2000/1/1:2020/1/1[pdat]) AND (english[Filter])) | 4th March 2022 | 170 |
| 4 | Cinhal | Search Alert: "(TX work related stress OR TX ( occupational stress or workplace stress or job stress ) OR TX ( wellbeing or well-being or well being ) OR TX ( occupational health or occupational wellbeing )) AND (S1 AND S2) Full Text; Published Date: 20000101-20221231 AND Apply equivalent subjects on 2022-03-15 07:28 AM" | 4th March 2022 | 67 |
| 5 | Proquest | Ebook Central ProQuest One Business Publicly Available Content Database These databases are searched for part of your query. Limited by: Date: From January 01 2000 to December 31 2021 Source type:Scholarly Journals Document type:Article Language:English (TI and AB combined 4055 results)  ti(Mindfulness based interventions) OR ti(mindfulness based stress reduction program) OR ti(Mindfulness programmes) OR ti(mindfulness training)Limits applied Databases: • Coronavirus Research Database • Ebook Central • ProQuest One Business • Publicly Available Content Database These databases are searched for part of your query. Limited by: Date: From January 01 2000 to December 31 2021 Source type: Scholarly Journals Document type: Article Language: English ab(Mindfulness based interventions) OR ab(mindfulness based stress reduction program) OR ab(mindfulness training) OR ab(mindfulness programmes)Limits applied Databases: • Coronavirus Research Database • Ebook Central • ProQuest One Business • Publicly Available Content Database These databases are searched for part of your query. Limited by: Date: From January 01 2000 to December 31 2021 Source type: Scholarly Journals Document type: Article Language: English | 4th March 2022 | 3897 |
